# Internet Gaming Disorder in adolescence: investigating profiles and associated risk factors

**DOI:** 10.1186/s12889-021-11394-4

**Published:** 2021-08-12

**Authors:** Mirna Macur, Halley M. Pontes

**Affiliations:** 1 Angela Boškin Faculty of Health Care, Spodnji Plavž 3, SI-4270 Jesenice, Slovenia; 2 Department of Organizational Psychology, Birkbeck, University of London, Malet Street, Bloomsbury, London, WC1E 7HX UK

**Keywords:** Internet Gaming Disorder, Behavioral addiction, American Psychiatric Association, Adolescents, Self-control, IGDS9-SF

## Abstract

**Background:**

Electronic gaming is a popular free-time activity and its deleterious effects have been considered by the American Psychiatric Association and World Health Organization. More recently ‘Gaming Disorder’ (GD) has been added to the 11^th^ revision of the International Classification of Diseases, while ‘Internet Gaming Disorder’ (IGD) remains as a tentative disorder in the 5^th^ revision of the Diagnostic and Statistical Manual of Mental Disorders. This study aimed to explore the characteristics of young gamers at risk for developing IGD.

**Methods:**

To achieve this, a quantitative and nationally representative study was conducted in primary schools in Slovenia with eighth grade as the primary sampling unit (*N* = 1071, Mean_age_ = 13.44 years, SD_age_ = 0.59). Psychometric testing assessing IGD was conducted to identify participants’ IGD risk levels and to compare ‘high risk gamers’, ‘low risk gamers’, and ‘non-gamers’ in relation to free-time activities, self-control, and parent-child relationship. A one-way ANOVA analysis was conducted with Games-Howell post-hoc test to compare the three groups of participants. Statistically significant IGD factors were then included in a multinomial logistic regression analysis to identify the most relevant predictors of IGD.

**Results:**

About 4.7% (*n* = 48) [95% CI: 3.4–6.0%] of Slovenian adolescents were found to be ‘high risk gamers’ when considering risk of IGD. These were mostly males (*n* = 42, 87.5%), and their preferred leisure activities involved more screen time activities (e.g., watching TV, playing video games, using social media). Moreover, ‘high risk gamers’ showed significantly lower levels of self-control compared to ‘low risk gamers’, and poorer understanding with their parents. Perceived satisfaction with life and mental health did not differ significantly between the three groups. The multinomial logistic regression identified four key predictors of IGD: male gender, gaming as a frequent free-time activity, attending music school or a choir and self-control.

**Conclusion:**

Public health measures should target adolescents at increased risk of developing IGD in early age because they are particularly drawn to excessive gaming behaviors and present greater IGD vulnerability.

## Background

Gaming is a popular and fast-growing leisure activity worldwide with the estimated number of gamers in 2020 being around 2.7 billion individuals [1]. Given how pervasive gaming is, particularly among younger individuals, concerns have emerged regarding disordered gaming behaviors, leading the American Psychiatric Association (APA) to include ‘Internet Gaming Disorder’ (IGD) within Section III of the fifth revision of the Diagnostic and Statistical Manual of Mental Disorders (DSM-5) as a tentative disorder requiring further research [2].

More recently, the World Health Organization (WHO) has decided to officially include ‘Gaming Disorder’ (GD) as a mental health condition within the latest (eleventh) revision of the International Classification of Diseases (ICD-11) [3, 4]. Table 1 illustrates the current conceptual approach to disordered gaming within the APA and WHO diagnostic framework.

**Table 1:** Definition of Internet Gaming Disorder (IGD) and Gaming Disorder (GD)

IGD definition and clinical criteria [2]:	GD definition and clinical criteria [3]:
*»Persistent and recurrent use of the internet to engage in games, often with other players,* *leading to clinically significant impairment or distress as indicated by five (or more) of the* *following in a 12-month period:* *Preoccupation with internet games. (The individual thinks about previous gaming activity or anticipates playing the next game; internet gaming becomes the dominant activity in daily life).* *Withdrawal symptoms when internet gaming is taken away. (These symptoms are typically described as irritability, anxiety, or sadness, but there are no physical signs of pharmacological withdrawal.)* *Tolerance—the need to spend increasing amounts of time engaged in internet games.* *Unsuccessful attempts to control the participation in internet games.* *Loss of interests in previous hobbies and entertainment as a result of, and with the exception of, internet games.* *Continued excessive use of internet games despite knowledge of psychosocial problems.* *Has deceived family members, therapists, or others regarding the amount of internet gaming.* *Use of internet games to escape or relieve a negative mood (*e.g.*, feelings of helplessness,* *guilt, anxiety).* *Has jeopardized or lost a significant relationship, job, or educational or career opportunity because of participation in internet games.*	A pattern of gaming behavior (“digital-gaming” or “video-gaming”) characterized within a 12-month timeframe by:
	*“impaired control over gaming, increasing priority given to gaming over other activities to the extent that gaming takes precedence over other interests and daily activities, and continuation or escalation of gaming despite the occurrence of negative consequences.*
	*For gaming disorder to be diagnosed, the behavior pattern must be of sufficient severity to result in significant impairment in personal, family, social, educational, occupational or other important areas of functioning and would normally have been evident for at least 12 months.”*

Previous research has reported significant variation in prevalence rates of disordered gaming across a wide range of studies. Griffiths et al. [5] found prevalence rates ranging from 0.2 to 34% and reported that disparities may be due to different sample sizes, distinct age range, sampling characteristics (general population vs. gamers only), type of gaming activity assessed, and psychometric test used to assess disordered gaming [6]. However, in representative samples of children and adolescents, previous findings reported that about 2% are likely to be affected by IGD [7], with a similar prevalence rate of 2.5% being found among Slovenian adolescents [8].

Most of the epidemiological findings have reported higher IGD prevalence rates among males in comparison to females [9–16]. Furthermore, in children and adolescent samples, IGD may be observed up to five times more often among males than females [7, 9, 14].

Disordered gaming has also been linked to several negative health-related outcomes and psychosocial detriments, such as increased stress, increased obesity, decreased job performance and/or job loss, decreased academic achievement, sleep abnormalities, relationship problems, depression, lower psychosocial wellbeing, and anxiety [7] alongside other similar addictive disorders involving excessive technology use [17]. Additionally, Severo et al. [18] recently found that increased depressive symptoms, poor sleeping, male gender, greater time spent gaming, and total free-time spent gaming are key IGD risk factors. In addition, other adolescent studies reported increased time spent gaming among disordered gamers [19], a phenomenon that is arguably reflected within the IGD definition itself through the operationalization of tolerance symptoms in IGD (i.e., “tolerance—the need to spend increasing amounts of time engaged gaming”) [2].

Recent longitudinal research carried out by Teng et al. [10] has examined the links between psychosocial wellbeing and IGD among older aged adolescents and emerging adults. The results of this study suggested that IGD is a maladaptive response leading to poorer psychosocial wellbeing, further showing that IGD is negatively associated with self-esteem and social support at the longitudinal level [10]. Similarly, the Slovenian representative study Health Behavior in School-Aged Children (HBSC) conducted in 2018 (among 11-, 13-, 15- and 17-year-old adolescents, *N* = 7449) reported higher odds for IGD among male adolescents (OR = 4.5), experiencing greater difficulties to communicate with their fathers (OR = 1.4), presenting with multiple health complaints more than once a week (OR = 1.8), engaging in bullying (OR = 2.3) or being bullied (OR = 1.3), feeling pressured with school work (OR = 1.4), and reporting feeling less supported by their friends (OR = 1.2) [14].

In relation to personality traits and other health-related factors, IGD has been shown to have associations with aggression and hostility, neuroticism, loneliness and introversion, avoidant and schizoid interpersonal tendencies, boredom inclination, social inhibition, sensation-seeking, diminished self-control and narcissistic personality traits, lower agreeableness, greater state and trait anxiety levels, lower self-esteem and emotional intelligence [20]. Moreover, loss of control is featured in the definition of IGD (“Unsuccessful attempts to control the participation in internet games”) [2] and has been systematically reported by other IGD studies [7, 21–24]. Relatedly, de Hesselle and colleagues have recently shown that lower levels of extraversion, agreeableness, and conscientiousness predict increased gaming time, with male gamers being more likely to spend greater time playing video games compared to female gamers [25].

Other studies reported important parental influence in IGD development, such as poor parental competence and supervision, inconsistent parental behavior and poor parental care [7]. Jeong et al. reported that conflict between parents resulted in greater IGD symptom-severity in children after 1 year [26]. A systematic review of family factors in adolescent IGD [27] reported four important factors: parent–child relationship, parent status (e.g., mental health, socioeconomic status), family environment (e.g., number of people living in a household) and parental influence on gaming (e.g., attitudes toward gaming, parental supervision). Parent-child relationships remain as a critical topic of several studies, which associate poorer quality relationships with increased severity of IGD [27].

### The present study

This study aims to explore key factors associated with IGD on a representative sample of Slovenian adolescents in terms of free-time activities, self-control, and quality of parental relationship. Based on the APA recommendations and previous research, participants endorsing at least five out of the nine IGD criteria (i.e., as assessed by the Internet Gaming Disorder-Short Form; IGDS9-SF) were classed as disordered gamers [28]. In this study, those who answered with either ‘often’ or ‘very often’ to at least five items of the IGDS9-SF were classed as ‘high risk gamers’ and were compared to ‘low risk gamers’ and ‘non-gamers’.

## Methods

### Sample and procedure

This study was part of the Slovenian project “For the Health of the Young”^1^ which was supported by Norway Grants. To achieve the study’s goals, a random sample of Slovenian primary schools was recruited through stratification according to the 12 Slovenian statistical regions and population density. Prior to collecting the data, we contacted school principals and invited them to participate in the study. In each school only one eighth grade class participated in the study. The study was then presented to teachers and students, and they were asked to participate after written parental informed consent had been provided. The data was collected using desktop computers (from April to May 2015) with the aid of trained staff supporting the participants.

In total, 1095 participants were initially recruited and cases with severe missing values (i.e., 10%) were excluded from the study. The data were weighted according to the 12 statistical regions of Slovenia to represent the general population of eighth graders. After cleaning the data, a final representative sample of the target population was obtained (*N* = 1071). Participants’ ages ranged from 12 to 16 years (Mean_age_ = 13.44 years; SD_age_ = 0.59). Of those, 89% were born in 2001, and gender distribution was relatively even (i.e., 50.2% male).

### Measures

Demographic variables consisted of gender, region, and age at the time of the study (calculated from year and month of birth). Gaming-related variables consisted of time spent gaming from Monday to Friday and separately for weekends (Saturdays and Sundays). Disordered gaming was measured with the IGDS9-SF [28] which contains a set of nine items reflecting the diagnostic criteria to asses IGD within the APA framework. All nine items can be responded to using a Likert scale ranging from 1 ('never') to 5 (' very often'), with greater scores indicating higher severity of disordered gaming. Psychometric evidence regarding the validity and reliability of the IGDS9-SF in Slovenian adolescents has been previously shown to be adequate [8]. We opted to utilize the IGDS9-SF within the present study as it is one of the most widely psychometric assessment tools in the assessment of IGD and its psychometric properties have been extensively investigated in cross-cultural research conducted across many countries and language, including but not limited to German [29], Polish [30], European and South American Portuguese [31, 32], Spanish [33], and Turkish [34]. In the present study, the IGDS9-SF presented with adequate internal consistency (Cronbach’s α = 0.924).

Independent variables consisted of self-control, which was measured with the following seven items [35, 36]: “*I wholeheartedly take part in exciting things if I have to take an examination tomorrow*”, “*I abandon a task once it becomes hard and laborious*”, “*I am apt to enjoy risky activities*”, “*I enjoy teasing and harassing other people*”, “*I feel like I am a ticking time bomb*”, “*I lose my temper whenever I get angry*” and “*I habitually don’t do my homework*”. Responses were measured on a Likert scale ranging from 1 (‘*strongly agree*’) to 5 (‘*strongly disagree*’). A Korean panel study that used this self-control assessment tool reported that self-control has been significantly associated with problematic online behaviors [36]. The Slovenian version of this measure used in the present study had adequate levels of internal consistency (Cronbach’s α = 0.825); in results section we present sum of all seven items reversed with values ranging from 7 (strong self control) to 35 (lowest possible self control).

Perceived life-satisfaction was measured by a single question asking participants to rate the following questions “*How satisfied are you with your life?*” using a Likert scale ranging from 1 (‘*completely dissatisfied’*) to 10 (‘*totally satisfied*’). Similarly, perceived mental health was measured with the following question “*How would you assess your mental health in general?*”, rated on a Likert scale ranging from 1 (‘*bad*’) to 5 (‘*excellent*’).

Parent-child relationship was assessed with four items measuring perceived quality in parental relationship [36, 37]. The scale comprised the following statements on shared activities and perceived emotional closeness: “*My parents and I try to spend much time together*”, “*My parents always treat me with love and affection*”, “*My parents and I have frequent conversations*”, and “*My parents and I understand each other well*”. Responses too all four items are given on a Likert scale ranging from 1 (‘*very untrue’*) to 5 (‘*very true’*). The Slovenian version of this measure used in the present study showed adequate levels of internal consistency (Cronbach’s α = 0.856).

Free-time activities were measured in relation to several activities that involved spending time with friends; watching TV; playing videogames on computer/tablet/phone; using social media; reading books; attending music school or a choir; doing sports activities. All free-time activities were rated using a Likert scale ranging from 1 (‘*never*’) to 5 (‘*very often/always*’).

### Statistical analyses

IBM SPSS Statistics 25 was used for the statistical analyses. Prior to conducting the analyses, three groups of participants were formed: ‘non-gamers’, which included participants who answered ‘*no*’ to the question: “*Did you play computer games in last 12 months irrespective of the device (computer, tablet, smartphone)?*”. Following this, participants who had reported playing video games in last 12 months were then classified as either ‘high risk gamers’ (i.e., those who answered ‘*often*’ or ‘*very often*’ to at least five items of the IGDS9-SF) or ‘low risk gamers’ (all the remaining gamers).

Differences between these three groups of participants in relation to free-time activities, perceived life satisfaction and mental health, self-control, and parent-child relationship were investigated with the help of a One-Way Analysis of Variance (ANOVA). For pairwise comparisons, Games-Howell post hoc tests were used due to the different sample sizes within each group [38]. Statistically significant pairwise comparisons were subsequently included into a multinomial logistic regression to further unravel key IGD predictors.

## Results

### Comparison of the three groups of participants

Based on the aforementioned operationalization, about 4.7% (*n* = 48) [95% CI: 3.4–6.0%] of the total sample was classified as ‘high risk gamers’ due to potentially meeting a positive IGD diagnosis. The most frequently endorsed IGD criteria were loss of control (criterion 4, *n* = 44), escapism (criterion 8, *n* = 44), and tolerance (criterion 3, *n* = 43). Participants classed as ‘high risk gamers’ were mostly male (87.5%, *n* = 42). The main characteristics of all three groups of participants are presented on Table 2.

**Table 2:** Characteristics of the three groups of participants

	Non-gamers	Low risk gamers	High risk gamers	Total
n	185	790	48	1023
Male (n, %)	45 (24.3%)	416 (52.7%)	42 (87.5%)	503
Female (n, %)	140 (75,5%)	374 (47.3%)	6 (12.5%)	520
Age (mean SD)	13.47 (0.62)	13.42 (0.58)	13.52 (0.57)	13.43 (0.59)
Time spent gaming Monday-Friday (hours/day) (mean SD)		1.47 (1.90)	4.42 (3.18)	
Time spent gaming Saturday-Sunday (hours/day) (mean SD)		2.18 (2.51)	6.77 (3.97)	

Age of participants across all three groups did not differ significantly (*F* = 1.154, *p* = 0.316). Furthermore, gender differed across the groups (*X*^2^ = 77.756, *p* < 0.001), with males prevailing among ‘high risk gamers’ (87.5%), whereas females prevailed among ‘non-gamers’ (75.5%). Average time spent gaming was significantly different among ‘high risk’ and ‘low risk gamers’ as ‘high risk gamers’ spent about 3 h longer gaming than ‘low risk gamers’ per weekday (*t*_[39.01]_ = − 5.637, *p* < 0.001), and approximately 4.5 h per day longer on weekends (*t*_[35.12]_ = − 6.697, *p* < 0.001).

Table 3 summarizes the findings regarding free-time activities of the three groups of gamers. As expected, statistically significant differences emerged between the three groups of participants in relation to the frequency of playing video games in their free-time with ‘high risk gamers’ reporting playing often and (almost) always, and ‘low risk gamers’ reporting playing sometimes. However the whole sample’s reported values for spending time with friends (Mean = 3.85, SD = 0.95) and sports’ activities (Mean = 3.77, SD = 1.21) were both higher than playing video games (Mean = 2.95, SD = 1.20), suggesting a preference for spending greater time engaged with non-digital activties.

**Table 3:** Analysis of variance of the three groups of participants regarding free-time activities, life satisfaction, mental health, parent-child relationship and self-control

	Non-gamers	Low risk gamers	High risk gamers	ANOVA		
	Mean (SD)	Mean (SD)	Mean (SD)	F	*p*	Post hoc
Free-time activities						
Time spent with friends	3.94 (0.95)	3.84 (0.92)	3.78 (1.32)	1.126	0.325	
Watch TV	2.96 (1.06)	3.10 (0.96)	3.39 (1.13)	3.792	0.023	
Play video games on computer/tablet/phone	1.02 (0.72)	3.12 (1.08)	4.27 (0.87)	112.456	< 0.001	c > b > a
Engage in social media	3.30 (1.34)	3.25 (1.25)	3.69 (1.38)	2.620	0.073	
Read books	2.50 (1.39)	2.38 (1.12)	2.03 (1.19)	3.134	0.044	
Attend a music school or a choir	2.19 (1.67)	1.78 (1.39)	2.10 (1.62)	6.603	0.001	a > b
Sports activities	3.61 (1.30)	3.80 (1.19)	3.80 (1.20)	1.863	0.156	
Perceived life satisfaction	7.69 (2.25)	8.07 (1.99)	7.29 (3.07)	4.842	0.008	
Perceived mental health	3.76 (1.11)	3.94 (1.04)	3.71 (1.55)	2.774	0.063	
Parent-child relationship: My parents						
sum of all parent-child items	15.56 (3.81)	15.63 (3.46)	14.07 (4.39)	4.199	0.015	
and I try to spend much time together	3.75 (1.09)	3.81 (1.02)	3.49 (1.33)	2.244	0.107	
always treat me with love and affection	3.61 (1.17)	3.64 (1.16)	3.29 (1.29)	1.949	0.143	
and I have frequent conversations	4.07 (1.05)	3.98 (1.01)	3.60 (1.37)	3.796	0.023	
and I understand each other well	4.11 (1.05)	4.21 (0.99)	3.72 (1.32)	5.648	0.004	b > c
Overall self-control level	18.25 (6.15)	18.56 (5.94)	25.49 (8.33)	28.496	< 0.001	a > c, b > c

Scales: free-time activities from 1(‘*never*’) to 5 (‘*almost always/always*’); perceived life satisfaction from 1 (‘*completely dissatisfied’*) to 10 (‘*totally satisfied*’); perceived mental health from 1 (‘*bad*’) to 5 (‘*excellent*’); parent-child relationship from 1 (‘*very untrue’*) to 5 (‘*very true’*): higher value means more favourable relationship and self-control as a sum of seven self-control variables reversed ranging from 7 (strong self control) to 35 (lowest possible self control)

^a^: Non gamers; ^b^: Low risk gamers; ^c^: High risk gamers; Post hoc: Games-Howell

Participants’ levels of satisfaction with their lives were relatively high (whole sample Mean = 7.97, SD = 2.11) and the assessment of their mental health state was very good (whole sample Mean = 3.89, SD = 1.08). The Games-Howell post hoc test did not show significant differences among the three groups of participants. On the whole, the total sample assessment of the relationship with their parents was high, with mean values ranging from 3.62 (SD = 1.17) to 4.17 (SD = 1.02), indicating rather favorable parent-child relationships among the participants. The best assessment on the whole sample was attributed to good understanding with their parents (Mean = 4.17, SD = 1.02), however there were significant differences between the ‘low risk gamers’ and ‘high risk gamers’ with the later reporting poorer understanding with their parents (Mean_LRG_ = 4.21, SD_LRG_ = 0.99, Mean_HRG_ = 3.72, SD_HRG_ = 1.32).

In relation to self-control, ‘high risk gamers’ showed significantly higher levels of self- control problems (Mean = 25.49, SD = 8.33) when compared to both ‘low risk gamers’ (Mean = 18.56, SD = 5.94) and ‘non-gamers’ (Mean = 18.25, SD = 6.15).

Effect sizes in the Table 3 are small; the exception is item “play video games” in their free-time, where eta squared is .189.

### Key predictors of ‘low risk gamers’ and ‘high risk gamers’ among adolescents

A multinomial logistic regression analysis was conducted as the dependent variable had three levels (i.e., ‘non-gamers’, ‘low risk gamers’, and ‘high risk gamers’). All the variables reported on Table 2 were added as independent variables to the regression model as they have shown in the previous analyses to exert important effects. Male gender (0-female; 1-male) was added, but not age as it did not differ across three groups, and time spent gaming (the two variables had missing values in the group of ‘non-gamers’).

The final model presented in Table 4 had a significant improvement in fit over the null model [*X*^2^(10) = 265.416; *p* < 0.001]. Likelihood ratio tests showed that only four independent variables contributed to the model. These variables were male gender, playing video games during free-time, attending music school or choir and overall self-control levels.

**Table 4:** Parameter estimates of the multinomial regression analysis

	Low risk gamers		High risk gamers	
	B (*p* value)	AOR (95%CI)^a^	B (*p* value)	AOR (95%CI)^a^
Intercept	−1.327 (0.041)		−11.068 (< 0.001)	
Male gender	0.610 (0.004)	1.841 (1.217–2.784)	1.989 (< 0.001)	7.308 (2.598–20.562)
Play video games on computer/tablet/phone	0.976 (< 0.001)	2.653 (2.163–3.255)	1.795 (< 0.001)	6.020 (3.946–9.183)
Attend a music school or a choir	−0.068 (0.260)	0.935 (0.831–1.051)	0.311 (0.015)	1.365 (1.064–1.751)
My parents and I understand each other well	0.086 (0.370)	1.090 (0.903–1.315)	0.001 (0.994)	0.994 (1.001–0.704)
Self-control	−0.007 (0.677)	0.993 (0.961–1.026)	0.106 (0.001)	1.112 (1.045–1.183)

^a^ The reference category is: non-gamers

The reference category in the model was ‘non-gamers’. Only two variables were significant for describing ‘low risk gamers’ (i.e., male gender and playing video games during free-time). For every unit of increase in frequency of playing video games during free-time, the odds of being classed as a ‘low risk gamer’ changes by 2.653. ‘Non-gamers’ and ‘high risk gamers’ comparison is more informative – four variables emerged as significant predictors of ‘high risk gamers’ (i.e., male gender, video-game playing, attending music school, and overall self-control levels). When frequency of playing video games during free-time increases by one unit, the odds of being classed as a ‘high risk gamer’ changes by 6.020. Moreover, when frequency of attending music school or choir increases by one unit, the odds change by 1.356. Finally, if overall self-control increases by a unit (i.e., meaning lower self-control levels), then the odds of being classed as a ‘high risk gamer’ increases by 1.112 (see Table 4).

## Discussion

Video games are one of the most popular leisure activities of children and adolescents worldwide, with dysregulated gaming being able to lead to addiction-like symptoms. A systematic literature review on IGD by Paulus et al. showed that IGD may be observed up to five times more often among male children than female children [7], and the present study showed that male gender was an important predictor of disordered gaming, a finding that was reported across several past studies [9–16, 19].

Moreover, free-time activities of ‘high risk gamers’ were mostly related to screen time activities than non-screen time activities when compared to ‘low risk gamers’ and ‘non-gamers’. Specifically, ‘high risk gamers’ were found to play more video games, watch more TV, and engage more in social media. Nevertheless, the majority of adolescents in that particular age had healthier habits of spending their free-time as they more frequently spent time with their friends and engaged more in sports activities than played video games. Surprisingly, the present study showed no statistically significant differences in regards to free-time sports activities between ‘high risk gamers’ and the two other groups of participants (‘low risk gamers’ and ‘non-gamers’) even though previous research has suggested that IGD is associated with obesity [20] and overweight in young adult females [39]. This study also found that time spent gaming was sigificantly greater for ‘high risk gamers’ both on weekdays and weekends in comparison to ‘low risk gamers’. Time spent gaming is an important predictor of IGD that is frequently reported in similar studies [19], as well as being reflected in the APA framework for IGD [2] via tolerance (i.e., the need to spend increasing amounts of time engaged in gaming).

There are several internal and external factors related to development of IGD [7], including self-control (internal) and parental influences (external), which were assessed in the present study. More specifically, we found that ‘high risk gamers’ presented with significantly lower levels self-control when compared to ‘low risk gamers’ and ‘non-gamers’. Several studies reported lower levels of self-control among heavy gamers [21–24] in addition to increased impulsivity [40–42].

Although self-control is an important aspect of the diagnostic criteria of IGD developed by the APA, a study by Pontes et al. [43] suggested that different IGD criteria may carry different diagnostic weight. Accordingly, ‘withdrawal’ (criterion 2) and ‘loss of control’ (criterion 4) have been reported as key factors in the etiology of IGD as together they may increase the likelihood of IGD by 77.77% [43]. However, answering ‘*very often*’ in the IGDS9-SF in relation to ‘withdrawal’, ‘loss of control’ and ‘negative consequences’ (criterion 9), may increase the likelihood of IGD by 26.66% [43]. Moreover, King et al. reported loss of control as one of the core symptoms of disordered gaming, together with withdrawal and conflict, and other symptoms being considered peripheral (i.e., not essential for the diagnosis of IGD) [44]. Self-control emerged as a statistically significant predictor for ‘high risk gamers’ also in our study.

There are additional external factors contributing to the development of IGD, such as parental influence and parental factors related to poor parental competence and supervision, inconsistent parental behavior, and poor parental care [7]. In our study we did not find important differences in terms of parent-child relationship between ‘high-risk gamers’ and the other two groups of participants, apart from the fact that ‘low risk gamers’ reported better understanding in the context of their parental relationships than ‘high risk gamers’.

Previous studies have also shown that conflict between children and their parents leads to higher levels of IGD after 1 year [26]. Similarly, disordered gaming symptoms have been found to be higher among adolescents presenting with lower commitment to family social activities, whereas important prevention strategies may focus on strong parent-child relationships [19]. A study among Korean adolescents [45] revealed that while father-child communication reduced risk of IGD, mother-child relationship did not, which may imply that fathers should make additional efforts in order to improve open and positive communication skills with their children [26, 45].

Additionally, the Slovenian HBSC study investigating the role of key IGD predictors among adolescents found higher odds for IGD among those who presented with greater difficulties communicating with their fathers [14]. Furthermore, a study by Koning et al. revealed that parenting practices among disordered adolescent gamers had bidirectional effects, with increased parent-adolescent communications about the internet predicting greater IGD symptoms among males, while greater IGD symptoms predicting more reactive rules among males and females. Displaying IGD symptoms appears to elicit ineffective parental responses, which may further exacerbate disordered gaming [45].

Our study also found that self-reported life satisfaction and mental health state were not statistically different among the three groups of participants even though IGD in adolescence has been reported to be related to several types of health problems, including mental health difficulties [13, 14, 46]. Since the present sample was relatively young, participants may not be experiencing intense and severe mental health problems yet, therefore the findings obtained may be justified on the basis of the samples’ current developmental stage. Mental health disorders are statistically more prevalent among adults, where gaming and internet overuse may be related to coping strategies related to stress, depression, suicidal ideation, and suicide attempts [47].

Present study revealed less IGD predictors than Slovenian HBSC study, although they were both conducted in representative adolescent samples. The 2018 HBSC study used nine IGD variables with yes/no scale of answers, where adolescents were classed as being ‘at risk’ if they had provided 5 or more ‘*yes*’ answers [14]. The proportion of 'at risk' participants within the HBSC study was therefore higher than in present study, where ‘at risk gamers’ were calculated when 5 or more 4 (‘*often*’) or 5 (‘*very often*’) answers to the nine IGDS9-SF items were provided. Another reason for different number of calculated IGD predictor was the broader range of health related variables in the HBSC study whereas our study was limited to few independent variables. Nevertheless, both studies facilitate future preventive initiative based on their findings. Gaming is rapidly growing [1] – since the data were collected in 2015, prevalence rates of IGD may be higher now. We believe this complex phenomenon should be investigated by interdisciplinary teams in future research so that collaborative actions can take place in order to mitigate the burden of disordered gaming among youth.

Potential limitations in the present study may relate to the low number of independent variables within the current survey as time for designing and conducting research was limited and scheduled at the beginning of the project. Due to ethical and financial constraints, the survey was not possible to be extended in order to collect multilevel data, therefore, this may constitute as a potential limitation in the present study. Moreover, the data was collected in 2015 and it is possible that different findings would have been found, especially at the epidemiological level, if the data collection was more recent. Additional potential limitations may be related to the self-report nature of the study and lack of an experimental design capable of generating causal findings. Despite these potential issues, the present study offers novel insights on IGD based on a relatively large national sample from Slovenia.

## Conclusion

Disordered gaming may elicit a wide range of health- and relationship-related problems. Younger individuals are particularly drawn to gaming, so helping youth struggling with gaming problems is key since most addictions seem to develop in early adulthood. The pathway to addiction is characterized by a change in emphasis from fun through losing control, to obsession [7], therefore public health measures should target ‘high risk gamers’ in early preventive initiatives.

The present study showed that ‘high risk gamers’ presented with lower levels of self-control. With parental influence being diminished at this age, prevention efforts must be established to target adolescents who may experience excessive gaming or IGD. Several countries do not currently offer treatment to individuals struggling with IGD, with some non-governmental organizations providing help to affected gamers. The recent preliminary recognition of IGD by the APA and official recognition of GD by the WHO fostered additional investigation and research in this field but also treatment efforts in many countries.

## Data Availability

The data set used and analyzed during the current study is available from the corresponding author on reasonable request.

## Abbreviations

IGD: Internet Gaming Disorder
APA: American Psychiatric Association
DSM-5: Diagnostic and Statistical Manual of Mental Disorders, fifth edition
WHO: World Health Organization
GD: Gaming Disorder
ICD-11: Eleventh revision of the International Classification of Diseases
IGDS9-SF: Internet Gaming Disorder Scale–Short-Form
HBSC: Health behavior in school-aged children

## Footnotes

1 https://eeagrants.org/project-portal/project/SI05-0007
